# Early Rising

**Published:** 1889-11

**Authors:** 


### Early Rising.

All this talk about early rising is moonshine. The habit of turning out of bed in the middle of the night suits some people; let them enjoy it. But it is only folly to lay down a general rule upon the subject.

Some men are fit for nothing all day after they have risen early every morning. Their energies are deadened, their imaginations are heavy, their spirits are depressed.

It is said you can work so well in the morning. Some people can, but others can work best at night; others, again, in the afternoon. Long trial and experiment form only the conclusive tests upon these points.

As for getting up early because Professor All-Gammon has written letters to the papers proving the necessity of it, let no one be goose enough to do it.

We all know the model man, aged eighty: "I invariably arise at five; I work three hours, take a light breakfast—namely, a cracker and a pinch of salt; work five hours more; never smoke, never drink anything but barely-water, eat no dinner, and go to bed at six in the evening."

If anybody finds that donkeyfied sort of life suits him, by all means let him continue it. But few people would care to live to eighty on these terms. If a man can not get all withered and crumpled up on easier conditions than those, it is almost as well that he should depart before he is a nuisance to himself and a bore to everybody else.

Schoolboys, and young people generally, ought to get up early, for it is found that nine-tenths of them can stand it, and it does them good.

But let no one torture himself with the thought that he could have been twice as good a man as he is if he had risen every morning at daylight. The habit would kill half of us in less than five years.

